# Hematopoietic Effects of *Angelica gigas* Nakai Extract on Cyclophosphamide-Induced Myelosuppression

**DOI:** 10.3390/plants11243476

**Published:** 2022-12-12

**Authors:** Mincheol Kang, Seojin Park, Yuseong Chung, Je-Oh Lim, Jae Seon Kang, Jun Hong Park

**Affiliations:** 1 Herbal Medicine Resources Research Center, Korea Institute of Oriental Medicine, Naju-si 58245, Republic of Korea; 2 Bio and Drug Discovery Division, Korea Research Institute of Chemical Technology, Daejeon 34114, Republic of Korea; 3 Department of Pharmacy, Kyungsung University, Busan 48434, Republic of Korea

**Keywords:** *Angelica gigas* Nakai, myelosuppression, cyclophosphamide, hematopoiesis

## Abstract

Myelosuppression is a major adverse effect of chemotherapy. With the increasing number of cancer patients worldwide, there is a growing interest in therapeutic approaches that reduce the adverse effects of chemotherapy. *Angelica gigas* Nakai (AGN) roots have been widely used in oriental medicine to treat blood-related diseases, including cancer. However, the effects of AGN on myelosuppression have not been studied. Here, we investigated the effects of AGN ethanol extract (AGNEX) on cyclophosphamide-induced myelosuppression. AGNEX treatment significantly decreased white blood cell levels while increasing red blood cell and platelet levels in the peripheral blood. It inhibited thymus and spleen atrophy. It also enhanced serum levels of interleukin (IL)-6 and tumor necrosis factor (TNF)-α. qRT-PCR results showed that AGNEX decreased the expression of IL-1b and stem cell factor (SCF) in the bone marrow (BM) while increasing the mRNA expression of IL-3 and IL-6 in the spleen. Although AGNEX did not significantly decrease apoptosis and cell cycle arrest in the BM and splenocytes, AGNEX plays a positive role in cyclophosphamide-induced myelosuppression. AGNEX administration increased BM cells in the femur while decreasing apoptotic BM cells. These findings suggest that AGNEX could be used to treat myelosuppression and as a combination therapy in cancer patients.

## 1. Introduction

Although developments in medical technology have improved the survival rate (70%) of cancer patients in Korea, it remains the leading cause of death [1]. According to the Korean Ministry of Health and Welfare, the incidence of cancer is on the rise due to aging, stress, etc., and if a person lives till their life expectancy of 83 years, the incidence rate of cancer is approximately 38%. Undoubtedly, anticancer drug research and development, as well as chemotherapy for cancer patients, continue to grow. Therefore, research on ancient herbal medicines to prevent and treat chemotherapy-induced myelosuppression is needed [2,3].

*Angelica gigas* Nakai (*A. gigas* Nakai, AGN) is a member of the Umbelliferae family whose roots have been widely used in oriental medicine [4]. *Angelica sinensis* and *Angelica acutiloba* have white flowers and are native to China and Japan, respectively, whereas AGN grows in Korea and blooms purple flowers (double-umbrella shape) in September [5,6]. The root of AGN used as a tonic (known as danggui) has been used in oriental medicine for its blood-replenishing properties, as well as the treatment of constipation and cancer. Numerous pharmacological studies on AGN extracts have been conducted. The primary components responsible for the medicinal effects of AGN are decursin (D) and decursinol angelate (DA), both of which are abundant in the root [7,8,9,10]. AGN extract (AGNEX) has been confirmed in several studies for its ability to cross the blood–brain barrier (BBB) and its therapeutic effect on brain damage and cancer [5,6,10]. According to studies conducted on the effect of AGN on hematopoiesis, administration of AGN in an anemia mouse model significantly improved serum erythropoietin concentrations [11]. AGN elevates glutamate metabolism to promote erythropoiesis, inhibit BM cell apoptosis, nourish the blood, and promote the synthesis of hematopoietic growth factor (HGF) and the proliferation of hematopoietic progenitor cells (HPC) [12,13,14,15]. However, only a few studies have shown that AGNEX has hematopoietic therapeutic effects in myelosuppressive mice. Therefore, studies on extraction methods to increase AGNEX purity and the evaluation of its pharmacological efficacy are currently underway.

Myelosuppression or bone marrow (BM) suppression is a decrease in the production of red blood cells (RBCs), white blood cells (WBCs), and platelets (PLTs) in the BM. Viral infections, radiation therapy, and chemotherapy are all potential causes of myelosuppression [16,17,18]. The loss of these vital blood cells can cause diseases including anemia, neutropenia, and thrombocytopenia, as well as symptoms such as fatigue, headache, chills, and excessive bleeding. Blood cells are constantly generated, differentiated, and eliminated in the body. Hematopoiesis is the process of producing blood cells and plasma, essential for maintaining homeostasis and overcoming myelosuppression. Hematopoiesis involves tissues such as the BM, spleen, and liver, as well as hematopoietic growth factors, including granulocyte-macrophage colony-stimulating factor (GM-CSF), stem cell factor (SCF), and interleukin (IL)-6 [17,19,20,21]. Because most cancer patients develop myelosuppression as a result of chemotherapy and/or radiotherapy, our understanding of myelosuppression and hematopoiesis must be expanded, and novel clinical approaches for treating myelosuppression should be investigated.

To investigate the effects of AGN, we generated a myelosuppressive mouse model using cyclophosphamide (Cy). The Cy-induced myelosuppression model is characterized by a disturbance in peripheral blood cells, hematopoietic-associated cytokines, inflammatory cytokines, the cell cycle, the thymus index, and the spleen index, leading to an increase in apoptosis [22]. Thymic and splenic tissues, as well as inflammatory cytokines such as IL-1β, IL-6, and tumor necrosis factor-alpha (TNF-α), are known to be involved in hematopoietic and immune function [19,21,23,24]. In vitro, cell cycle and apoptosis were assessed using K562 cells, and in vivo, indicators of myelosuppression such as peripheral blood cells, body weight, spleen and thymus organ index, tibia and femur histopathology, cell cycle, apoptosis, inflammatory cytokines, and expression of hematopoiesis-related genes were assessed. Our findings indicated that AGNEX aided in the rapid recovery of myelosuppressive symptoms and can therefore be used as a therapeutic agent or adjuvant for myelosuppression, an adverse effect of cancer chemotherapy.

## 2. Results

### 2.1. Characteristics of Myelosuppressive Mice and the Effect of AGNEX in Peripheral Blood

**Figure 1:**
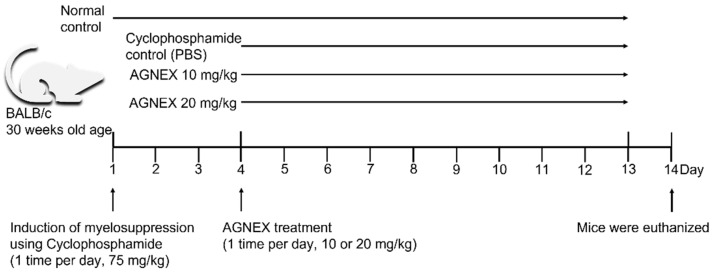
The experimental scheme used to induce myelosuppression and i.p. injection of AGNEX.

**Figure 2:**
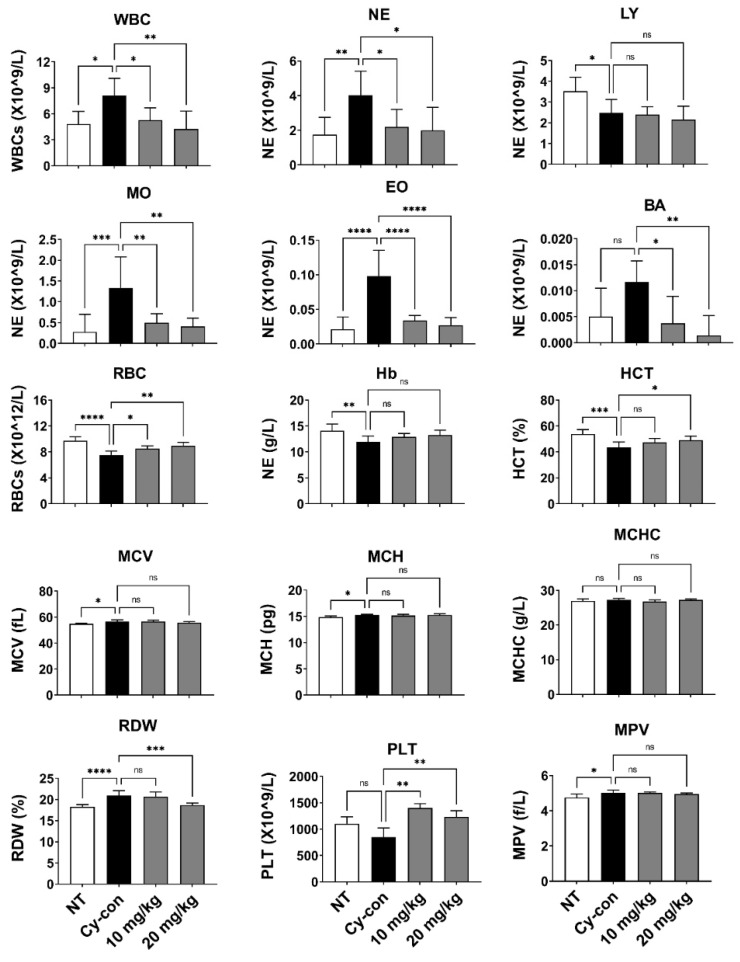
Effect of AGNEX on peripheral complete blood count (CBC). On day 14, CBC analysis of mouse peripheral blood was performed with HEMAVET. White blood cell (WBC), neutrophil (NE), lymphocyte (LY), monocyte (MO), eosinophil (EO), basophil (BA), red blood cell (RBC), hemoglobin (Hb), hematocrit (HCT), mean corpuscular volume (MCV), mean corpuscular hemoglobin (MCH), mean corpuscular hemoglobin concentration (MCHC), red cell distribution width (RDW), platelet (PLT), and mean corpuscular volume (MPV). Values represent the mean ± SD. ns: not significant, * *p* < 0.05, ** *p* < 0.01, *** *p* < 0.001, **** *p* < 0.0001, vs. Cy-con.

Myelosuppression model mice were prepared by injecting Cy intraperitoneally (i.p.). An untreated group (NT) was used as a negative control group, and a mouse injected with Cy was used as a positive control group (Cy-con). AGNEX was administered intraperitoneally at a concentration of 10 or 20 mg/kg to confirm its efficacy in model animals. On day 14, the composition of whole blood cells was compared using peripheral blood (Figure 1). The number of WBCs increased in the myelosuppression model mice (Cy-con) compared to the NT group, according to the complete blood count (CBC) measurements taken with a hematological analyzer. Cy-induced myelosuppression model mice are known to have increased WBCs [25]. Interestingly, in the groups of mice administered AGNEX, the number of WBCs of all types decreased, except for lymphocytes (Figure 2). Although the difference in hemoglobin (Hb) levels was unaffected, AGNEX restored the number of RBCs and PLTs in the peripheral blood to normal. Furthermore, the high concentration of AGNEX (20 mg/kg) treatment reduced the percentage of red cell distribution width (RDW). These results indicated that AGNEX was effective in decreasing WBCs and restoring RBCs and PLTs to their normal ranges in the peripheral blood of model mice with myelosuppression.

### 2.2. Effect of AGNEX on the Thymus and Spleen

**Figure 3:**
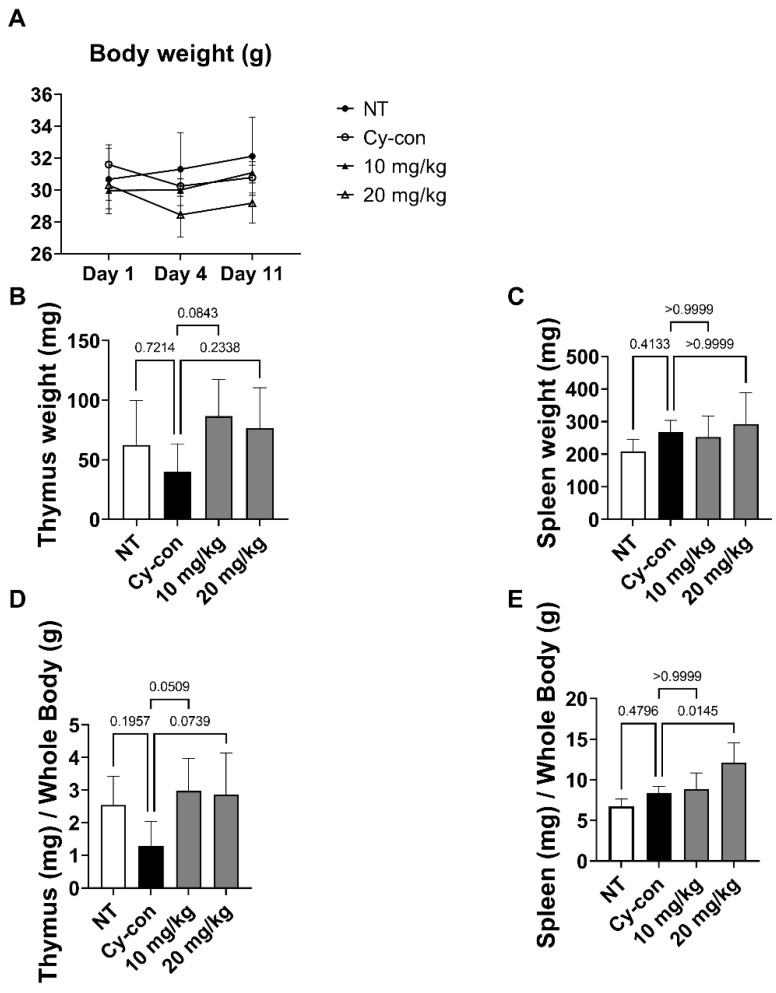
Effect of AGNEX on the body, thymus, and spleen weights in myelosuppressive mice. (**A**) Mice were weighed at three points during the experiment. (**B**,**C**) Organ weights of mice were measured on the last day of the experiment. (**D**,**E**) Organ index data were obtained by dividing the organ weight by body weight.

In the hematopoietic system, hematopoietic stem cells (HSCs) differentiate in the BM and migrate to the thymus and spleen as they mature. Thymus and spleen size are also indicators of hematopoiesis [21,25]. We measured the weight of organs through dissection and calculated the index by dividing the weight of each organ by the body weight (Figure 3). The thymus index decreased in the Cy-con group but was insignificant, and AGNEX alleviated hematopoietic atrophy caused by Cy. Furthermore, the 20 mg/kg AGNEX group had a higher spleen index compared to the NT and Cy-con groups.

### 2.3. Correlation between AGNEX and Hematopoietic Factors

**Figure 4:**
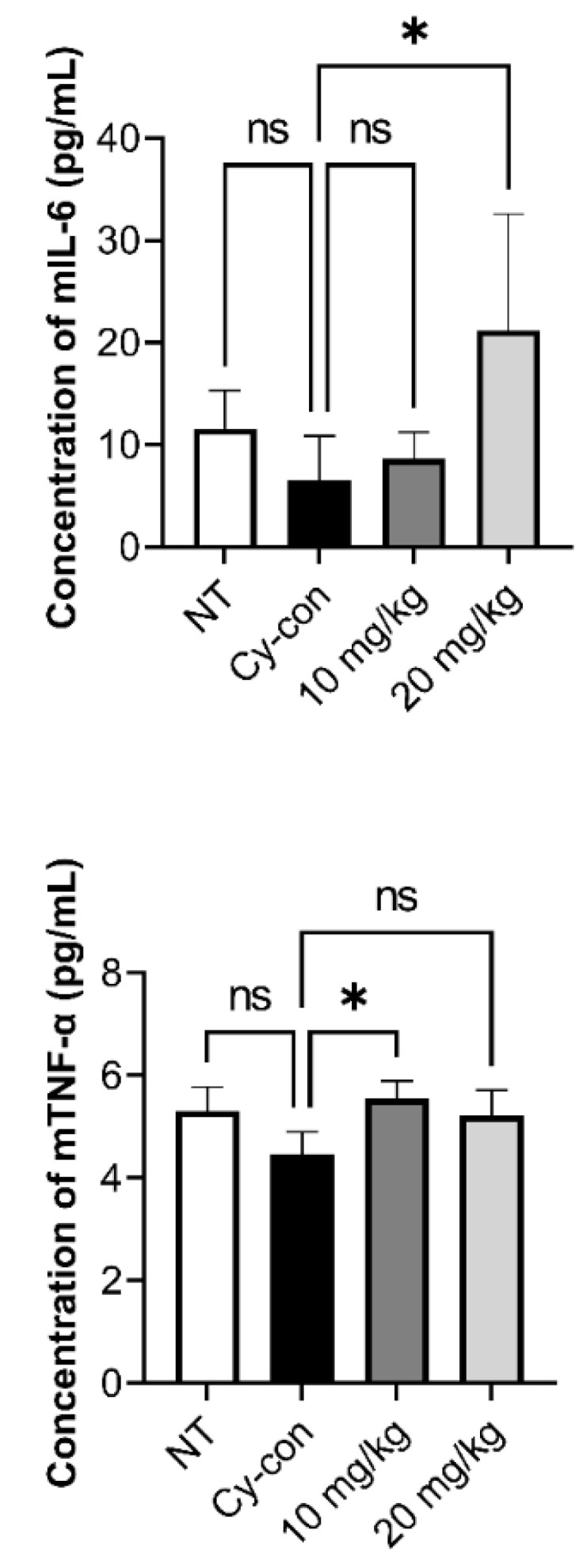
Effect of AGNEX on cytokine levels in myelosuppressive mice. On day 14, serum hematopoietic cytokines were analyzed by ELISA. Interleukin-6 (IL-6) and tumor necrosis factor-alpha (TNF-α). Values represent the mean ± SD. ns: not significant, * *p* < 0.05, vs. Cy-con.

**Figure 5:**
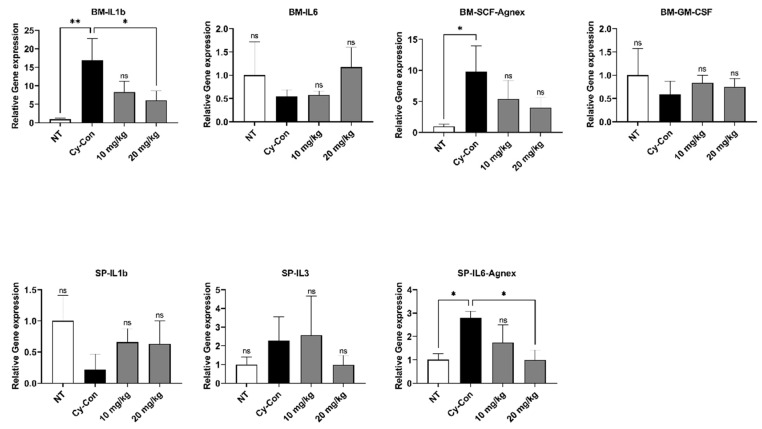
Effect of AGNEX on the mRNA level of BMSC (BM) and spleen (SP). On day 14, hematopoietic-related genes were analyzed in BM and SP by qRT-PCR. Interleukin-1 beta (IL-1β), IL-3, stem cell factor (SCF), and granulocyte monocyte colony-stimulating factor (GM-CSF). Values represent the mean ± SD. ns: not significant, * *p* < 0.05, ** *p* < 0.01, vs. Cy-con.

Many factors influence the differentiation and maturation of HSCs in hematopoiesis. In particular, tumor necrosis factor-alpha (TNF-α), is one of the pro-inflammatory cytokines that are known to mediate HSC survival, bone marrow regeneration, and lymphoid progenitor cell differentiation. Factors such as interleukin-1 beta (IL-1β), IL-3, IL-6, granulocyte-macrophage colony-stimulating factor (GM-CSF), and stem cell factor (SCF) are involved in the maturation and differentiation of myeloid progenitor cells [26,27,28]. Blood was collected from mice via submandibular blood sampling, and ELISA was performed after serum separation to determine the concentrations of IL-6 and TNF- in the blood (Figure 4). Furthermore, qRT-PCR analysis was carried out to confirm the expression patterns of various hematopoietic factors in the BM and spleen (Figure 5). The Cy-con group showed a similar level of IL-6 as the NT group, but the concentration of IL-6 increased significantly when 20 mg/kg of AGNEX was administered. TNF-α levels were higher, especially, when 10 mg/kg was administered to model mice.

The mRNA expression of hematopoiesis-related genes was analyzed in the spleen and BM. The IL-1b and SCF levels in the BM from AGNEX-treated mice were higher than those of the control group. Additionally, the high-dose-treated group tended to have elevated (but not significant) mRNA expression of IL-6 in the BM, but IL-6 reduced significantly in the spleen. No significant difference existed between the control and AGNEX-treated groups; however, the AGNEX-treated mice group exhibited a tendency for higher hematopoiesis-related gene expression than the control group. These findings suggest that AGNEX may aid in hematopoiesis in mouse models of myelosuppression. Our data indicate that AGNEX has a positive effect on the expression of factors essential for hematopoiesis.

### 2.4. Confirmation of Cell Proliferation and Viability of BM and Splenocytes

Cy, a cytotoxin, induces apoptosis and cell cycle arrest [29,30,31]. Cy-induced myelosuppression was accompanied by increased apoptosis and decreased cell cycle, thymus index, and spleen index [22]. After administration of AGNEX to myelosuppression model mice, we carried out flow cytometry experiments to analyze changes in the cell cycle and apoptosis of BM and splenocytes. After 10 days of Cy injection, there was no difference between groups in BM cell apoptosis (Figure S1). In the case of splenocytes, the Cy-con group had a greater proportion of dead cells (7-AAD+/Annexin V+) than the NT group. However, no significant difference was found between the AGNEX-administered and the Cy-con groups. To measure the cell cycle, BrdU (2 mg dissolved in 200 μL PBS) was injected intraperitoneally 12 h before dissection, and the cell cycle was analyzed using an anti-BrdU antibody and DAPI. When AGNEX was administered at a dose of 10 mg/kg, the percentage of splenocytes (Figure S2) and BM cells (Figure S3) in the G2/M phase increased marginally compared to the Cy-con group. Furthermore, the increased S phase and the decreased G2/M phase in the Cy-con group in comparison to the NT group indicate that cell cycle arrest has occurred. Even though splenocyte apoptosis increased in the Cy-injected groups, it was found that cell cycle arrest in BM and splenocyte was minutely alleviated when AGNEX at 10 mg/kg concentration was administered. As shown in the Supplementary Data, there were no significant differences between the Cy-con and AGNEX groups.

### 2.5. Validation of the AGNEX Efficacy in Erythroleukemia-Type Cells

K562 is a patient-derived erythroleukemia-type immortalized cell line. In the previous studies involving Cy and K562 cells, Cy exhibited cytotoxicity to deplete glutathione in K562 cells and was used in vitro to eliminate leukemia cells [32,33]. We evaluated the efficacy of AGNEX on Cy-induced apoptosis in K562 cells. AGNEX was added to the growth medium containing 1% DMSO at varying concentrations of 0, 0.01, 0.1, 1, and 10 µg/mL. The apoptosis of K562 cells was also measured by combining various concentrations of AGNEX with Cy at 3.5 mg/mL (Figure S4). The result confirmed the anti-apoptotic activity of AGNEX, as the proportion of dead cells (7-AAD+/Annexin V+) showed a decreasing trend. To investigate the effect of AGNEX on proliferation, the cell cycle of K546 cells cultured in the presence of varying concentrations of AGNEX was examined using an anti-BrdU antibody (Figure S5). Cell cycle analysis revealed that the AGNEX additive slightly increased the G2/M phase compared to the control (Figure S5). Although AGNEX cannot significantly reduce apoptosis and cell cycle arrest in K562, it has a protective effect against Cy-induced apoptosis in K562 cells. As shown in Supplementary Data, there were no significant differences between the Cy-con and AGNEX groups.

### 2.6. Validation of the AGNEX Ability to Recover and Differentiate in the Femoral Bone Marrow

**Figure 6:**
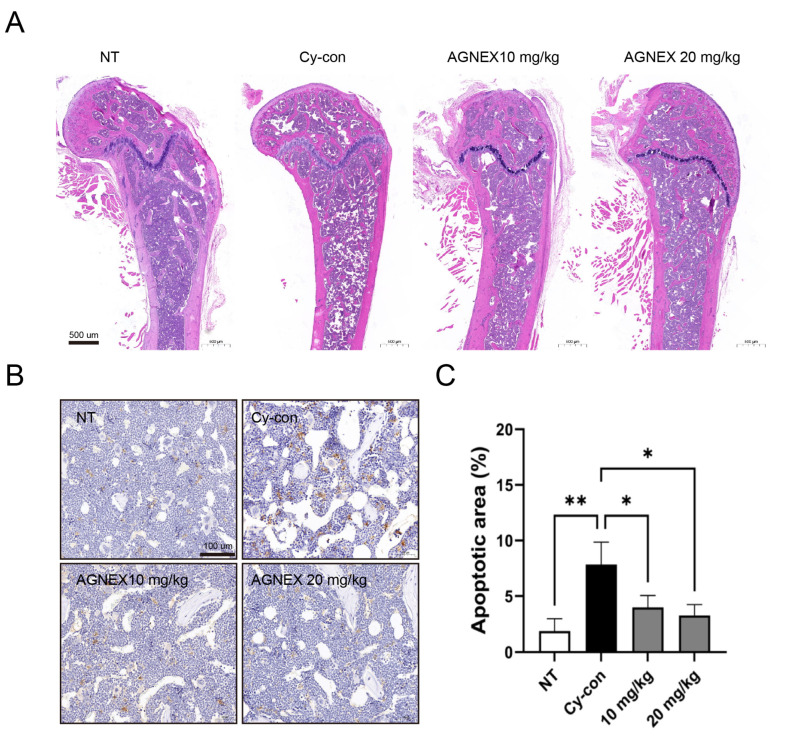
Effect of AGNEX on bone marrow tissue. Histological analysis was performed by routine and TUNEL staining. (**A**) The density of femoral bone marrow cells was confirmed through routine staining. (**B**,**C**) Apoptotic cells were detected by TUNEL assay. Values represent the mean ± SD. * *p* < 0.05, ** *p* < 0.01, vs. Cy-con.

When Cy is injected into mice, it kills BM cells and causes the mice to develop immunosuppressive symptoms. Histological analysis was performed to validate the potential of AGNEX as a therapeutic agent. H&E staining of the femur confirmed that the number of BM cells was lower in the Cy-con group compared to the NT group (Figure 6A). We confirmed that the BM cells decreased by Cy were recovered from the femur by AGNEX treatment. In addition, fewer apoptotic cells were detected in the AGNEX group than in the Cy-con group (Figure 6B,C). We demonstrated through histological analysis that AGNEX was effective for BM cell proliferation not only in vitro but also in vivo.

## 3. Discussion

**Figure 7:**
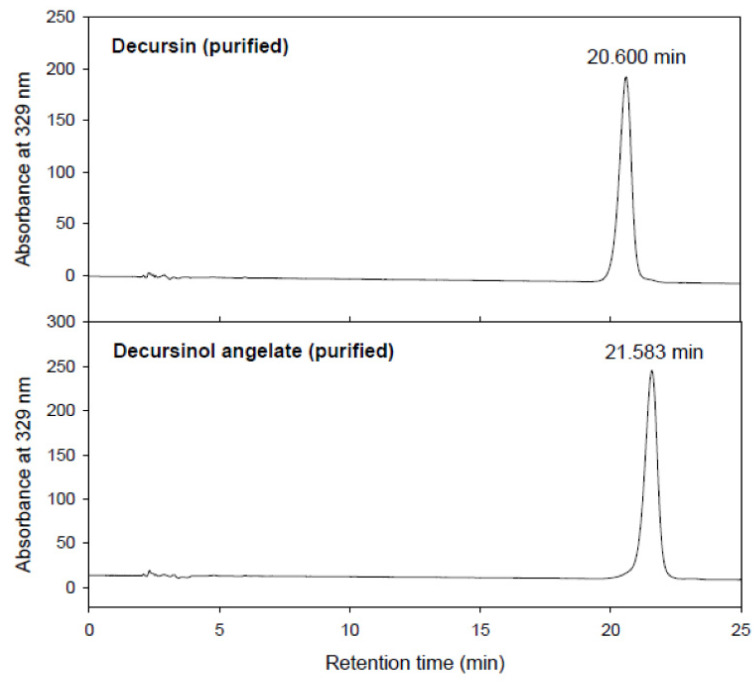
Detection of decrusin and decursinol angelate using high-performance liquid chromatography.

AGNEX contains essential oil and polyacetylene, as well as six abundant pyranocoumarins, including D, DA, decursinol, nodakenin, marmesin, and demethylsuberosine [34]. Zhang et al. demonstrated that among the six pyranocoumarins in AGN extracts, the quantity of major compounds (D, DA, and decursinol) in dried AGN roots was approximately 8.2% [35]. The HPLC chromatogram of the major compounds of AGN is presented in Figure 7, with the retention times for DOH, D, and DA being 4.79, 20.60, and 21.58 min, respectively. Previously, we found that the quantity of D/DA was 780.5 ± 0.02 mg/g. However, this amount corresponds to 78% of the constituents contained in the AGNEX [36]. It is important to obtain high-purity D/DA in AGNEX to prevent adverse effects, such as hypertension [37].

WBCs, a part of the body’s immune system, help fight infections and other diseases. According to previous studies, the increase in WBCs in the peripheral blood of model animals with myelosuppression is mobilized from the BM. WBCs mobilized by transient events do not play an immune role [25,38]. In our study, the number of WBCs in the AGNEX group was comparable to that in the NT group. It suggested that AGNEX may attenuate the symptoms of myelosuppression. The CBC test confirmed an increase in RBCs and PLTs. The ELISA and qRT-PCR results demonstrated the role of AGNEX in nourishing blood in vivo. AGNEX has also been found to be useful as an adjuvant in diseases such as chronic bleeding and anemia.

The spleen purifies the blood and produces blood cells, and the thymus is known to be an organ for the maturation of immune cells. Therefore, both the thymus and the spleen play an important role in the hematopoietic process [39,40,41]. Our findings suggest that AGNEX induced an increase in the size of the spleen and atrophied the thymus. These changes indicate that AGNEX plays a positive role in the maturation and differentiation of immune cells and circulating blood. Therefore, AGNEX could be used as a tonic in the treatment of radiotherapy- and chemotherapy-induced myelosuppression.

Various factors are involved in the hematopoietic process [26,27,28,42]. We confirmed that serum IL-6 and TNF-α levels were increased by AGNEX. In qRT-PCR results, although there were no significant differences in IL-6, SCF, and GM-SCF levels in BM between the Cy-con and AGNEX-treated group, IL-1β levels were significantly reduced in the high-dose-AGNEX-treated group (20 mg/kg). The expressions of IL-3 and IL-6 decreased significantly in the spleen following treatment with AGNEX. The alternation in these gene expression patterns might induce faster recovery from myelosuppression. Furthermore, the increase in PLTs and RBCs in the AGNEX groups compared to the Cy-con group in the CBC test may be due to these factors. Increased factors may promote the differentiation of common myeloid progenitor cells and influence their differentiation into megakaryocytes and erythrocytes. Histological analysis confirmed that the BM cells were recovered from the femur using AGNEX. We also found that the number of cells in the growth plate increased in the AGNEX groups compared to the Cy-con group. As one of the effects of AGNEX, it can be inferred that BM-derived mesenchymal stem cells were rapidly introduced and differentiated in the growth plate. Our histological findings suggest that AGNEX has therapeutic potential for diseases, such as osteoporosis and growth disorders. In the subsequent study, we aimed to validate the direction of AGNEX differentiation in cells using a hematological analysis method.

Cy is known to induce apoptosis and cell cycle arrest in myelosuppressive mice [22,30]. Our data indicated that there was no significant difference in the survival rate of BM cells in each group, but Cy increased the proportion of dead cells in splenocytes. In addition, it was confirmed that Cy caused severe cell cycle arrest in the S phase, thereby reducing the G2/M phase proportion. Our findings revealed that AGNEX promotes cell proliferation in vivo by abrogating the stagnant cell cycle. As demonstrated by the efficacy test, AGNEX had a positive effect on cell viability and proliferation of K562 cells. The reasons for the slightly different efficacy results of AGNEX in animal and cell studies include: animal experiments have more parameters and limitations than cell experiments; there are more variables in animal experiments than in cell experiments [43,44,45]; and different periods of administration. We have been administering AGNEX to myelosuppression model animals for a long time. Following AGNEX administration for 10 days, the mice may appear to have recovered from Cy-induced damage. In the next study, we intend to administer AGNEX to mice for a shorter period.

Taken together, this study confirmed that AGNEX derived from natural sources is worthy of in-depth research as a nutritional supplement for immune enhancement and as an adjuvant for preventing and treating myelosuppression in patients undergoing chemotherapy.

## 4. Materials and Methods

### 4.1. Preparation of Angelica gigas Nakai Extract (AGNEX)

The raw material for AGNEX was collected from the herbarium of Kyungsung University. The extraction process was carried out in accordance with our previous study with slight modifications [46]. Briefly, dried AGN was pulverized using a grinder machine (250G New Type Pulverizing Machine, Model RT-N04-2V, Taiwan) and the powder was sieved through a 60-mesh sieve to ensure uniform particle sizes. AGN powder was mixed with a 5-fold volume of 95% ethanol, followed by a 3 h incubation at 40 °C. The mixture was then filtered through a housing filter (35 µm pore size), a 2-fold volume of water was added, and the mixture was centrifuged at 10,000 rpm for 15 min, followed by pH adjustment to 5.0. The precipitate was dissolved in a 5-fold volume of 70% ethanol and applied at 20 Brix. The quantitative determination of decursin and decursinol angelate from AGN was carried out using the Agilent 1100 HPLC system (Santa Clara, CA, USA) equipped with a ZORBAX SB-C18 analytical column (250 mm, 4.6 mm, 5 µm; Agilent, Santa Clara, CA, USA). The purity of decursin and decursinol angelate of AGNEX was approximately 78% purity. Acetonitrile and 0.1% formic acid (70:30, *v*/*v*%) were used as the mobile phase. The absorbance was detected at 329 nm using a UV monitor (Photo-Diode Array UV monitor, Agilent, Santa Clara, CA, USA) (Figure 7).

### 4.2. Animal Experimental Design

In this study, 30-week-old BALB/c strain mice were used. Mice were housed in conventional laboratory cages at 24 ± 1 °C, 50 ± 5% humidity, and a 12 h alternate dark/light cycle (lights on at 7 AM). The normal control (NT) group did not receive any injections until the day of sacrifice. Cy (75 mg/kg, PHR1404-1G, Sigma-Aldrich, St. Louis, MO, USA) was intraperitoneally injected into the mice once a day for 3 days to induce myelosuppression in the three groups (Figure 1). Cy was dissolved in 1% dimethyl sulfoxide (DMSO, D8418, Sigma-Aldrich, St. Louis, MO, USA) in phosphate-buffered saline (PBS, 10010-023, Gibco, NY, USA). Mice were intraperitoneally injected with AGNEX (10 or 20 mg/kg) once a day for 10 days to confirm its efficacy. As a vehicle, 1% DMSO in PBS was injected into the Cy control group (Cy-con). Body weight was measured on days 1, 4, and 11, and the mice were euthanized on day 14 using CO_2_ gas for subsequent experiments. A total of 32 mice were used in this study, with 8 mice in each group (n = 8). All animal experiments were carried out in accordance with the guidelines for animal experimentation and were approved by the Animal Ethics Committee of Kyungsung University (approval number: research-21-022A).

### 4.3. Analysis of Mouse Peripheral Blood

Hematological analysis was performed on blood samples collected in EDTA-treated tubes (BD Microtainer^®^ 365974, BD, Franklin lakes, NJ, USA) using capillary tubes (2501, Kimble Chase, Vineland, NJ, USA). Anticoagulated blood was processed to determine a complete blood count (CBC) using a HEMAVET^®^ 950FS hematological analyzer (Drew Scientific, Miami Lakes, FL, USA), according to the manufacturer’s instructions.

### 4.4. Histological Analysis

For histological analysis, femur tissues were fixed in 10% neutral buffered formalin (NBF) and decalcified with a decalcifying solution (D0818, Sigma-Aldrich, St. Louis, MO, USA). The sections were then embedded in paraffin and sectioned at a thickness of 4 μm on each slide. The histology of the femur was assessed using hematoxylin and eosin (Routine, H&E) staining. The TUNEL assay was performed according to the manufacturer’s guidelines (Promega, Madison, WI, USA). Slides were examined and photographed using PANNORAMIC DESK II DW (3DHISTECH, Budapest, Hungary). The apoptotic area was calculated using the ImageJ software (version 1.41).

### 4.5. Quantitative Real-Time Polymerase Chain Reaction (qRT-PCR)

Total ribonucleic acid (RNA) was isolated from mouse splenocytes and BM cells using the easy-spin^TM^ (DNA-free) total RNA extraction kit (17221, iNtRON Biotechnology, Seongnam-si, Republic of Korea). Complementary deoxyribonucleic acid (cDNA) was synthesized from RNA using the iScript^TM^ cDNA synthesis kit (1708891, Bio-Rad, Hercules, CA, USA), according to the manufacturer’s instructions. For cDNA synthesis, a C1000 Touch^TM^ Thermal Cycler (Bio-Rad, CA) was used. RNA and cDNA concentrations were evaluated by spectrophotometry using a DS-11 Spectrophotometer (DeNovix Inc., Wilmington, DE, USA).

We used Power SYBR^TM^ Green PCR Master Mix (4367659, Applied Biosystems^TM^, ThermoFisher Scientific, Waltham, MA, USA) reagent for qRT-PCR, and analysis was performed using a CFX96^TM^ Real-Time System machine and Bio-Rad CFX Maestro software (Bio-Rad, CA). The cycling conditions were 95 °C for 5 min, followed by 40 cycles of 95 °C for 15 s, and 60 °C for 1 min. Transcript levels were calculated relative to the NT group, and the relative fold inductions were calculated using the 2^−ΔΔCt^ algorithm. Each set of primers shown in Table 1 was used for qRT-PCR [21], and glyceraldehyde 3-phosphate dehydrogenase (GAPDH) was used as an internal quantitative control.

### 4.6. Enzyme-Linked Immunosorbent Assay (ELISA)

Serum cytokine levels were quantified using a commercially available enzyme-linked immunosorbent assay kit. A blood sample was collected by the submandibular blood collection method using a heparinized capillary tube (2501, Kimble Chase, Vineland, NJ, USA), and serum was obtained using a serum separation tube (BD Microtainer^®^ 365974, BD, Franklin Lakes, NJ, USA). The concentrations of IL-6 and TNF-α were measured using ELISA kits (M6000B, and MTA00B, R&D Systems, Minneapolis, MN, USA), according to the manufacturer’s instructions. The absorbance of each well was determined using a SpectraMax i3x microplate reader and SoftMax Pro 7.0.3 (Molecular Devices, San Jose, CA, USA) set at 450 nm and 570 nm.

### 4.7. Statistical Analysis

Data were obtained from at least three independent experiments. A one-way ANOVA was performed using GraphPad Prism 9 (GraphPad Software, San Diego, CA, USA). The mean of each column was compared to the mean of the Cy-Con column. Multiple-comparison correction was used for Bonferroni hypothesis testing. Results are presented as mean ± standard deviation (SD) and significance was set at: *, *p* < 0.05; **, *p* < 0.01; ***, *p* < 0.001; and ****, *p* < 0.0001.

## Figures and Tables

**Table 1:** Primers used for qRT-PCR.

Gene	Primer Sequence	
murine *GAPDH*	Forward	: 5′-AGGTCGGTGTGAACGGATTTG-3′
	Reverse	: 5′-TGTAGACCATGTAGTTGAGGTCA-3′
murine *IL-1β*	Forward	: 5′-GCTACCTGTGTCTTTCCCGT-3′
	Reverse	: 5′-CGTCACACACCAGCAGGTTA-3′
murine *IL-3*	Forward	: 5′-GGTTCTTGCCAGCTCTACCA-3′
	Reverse	: 5′-GGTATCCCGGCCACTGATTG-3′
murine *IL-6*	Forward	: 5′-GTGGCTAAGGACCAAGACCA-3′
	Reverse	: 5′-TAACGCACTAGGTTTGCCGA-3′
murine *SCF*	Forward	: 5′-ATGTTCCCCGCTCTCTTTGG-3′
	Reverse	: 5′-GTGTGGCATAAGGGCTCACT-3′
murine *GM-CSF*	Forward	: 5′-GGCCATCAAAGAAGCCCTGA-3′
	Reverse	: 5′-TGGTGAAATTGCCCCGTAGA-3′

## Data Availability

Not applicable. All data generated or analyzed in this study are included in this published article.

## Supplementary Materials

The following supporting information can be downloaded at: https://www.mdpi.com/article/10.3390/plants11243476/s1, Figure S1: Effect of AGNEX on the induction of apoptosis in the BM cells and splenocytes (spleen) using cyclophosphamide; Figure S2: Confirmation of hematopoiesis by cell cycle analysis in splenocytes; Figure S3: Confirmation of hematopoiesis by cell cycle analysis in the BM; Figure S4: Effect of AGNEX on the induction of apoptosis in K562 cells using cyclophosphamide; and Figure S5: Cell cycle analysis of K562 cells cultured with varying concentrations of AGNEX.
