# Supplementary material for: Hematopoietic Effects of Angelica gigas Nakai Extract on Cyclophosphamide-Induced Myelosuppression
Source: Plants (Basel). 2022 Dec 12;11(24):3476. doi: 10.3390/plants11243476 (PMC9781469; doi:10.3390/plants11243476)
Supplement: Supplementary file 1 [file plants-11-03476-s001.zip › plants-2019854-supplementary.pdf]

Supplementary Material and Methods

Culture of K562 cells

The K562 cell line was obtained from the Korean Cell Line Bank (KCLB), Korea. K562 lymphoblastic cells were isolated from the bone marrow (BM) of chronic myelogenous leukemia patient and cultured in RPMI 1640 medium (Gibco, NY, 11875-093) supplemented with 10% heat-inactivated fetal bovine serum (Gibco, NY, 10082-147) and 1% antibiotic-antimycotic [100X] (Gibco, NY, 15240-062) in a humidified atmosphere with 5% CO_2 at 37°C. Subcultures (<13 passages) were cultured in T-75 flasks (Nunc™ Thermo Fisher Scientific, MA, 156499), and 1×10^6 cells were seeded into 6-well plates (Thermo Fisher Scientific, MA, 140675) for flow cytometry.

Flow cytometry analysis

Single splenocyte suspensions obtained from mice were prepared by treating them with Ammonium-Chloride-Potassium (ACK) Lysing Buffer (Gibco, NY, A1049201) for 5 min at room temperature. BM cells were extracted from the tibia and femurs of mice using Hank's Balanced Salt Solution (HBSS, Gibco, NY, 14170-112), washed with PBS, and then used for the experiments. K562 cells cultured under various conditions were harvested at 500 x*g* for 5 min using a centrifuge and used for flow cytometry analysis. Bromodeoxyuridine (BrdU, 2 mg dissolved in 200 μl PBS) was injected i.p. 12 h before mouse sacrifice to confirm the cell death. K562 cells were treated and cultured in BrdU (1 mM per mL of culture medium) for 45 min. Cells were stained using the Apoptosis, DNA damage, and Cell Proliferation kit and the PE Annexin V Apoptosis detection kit I (BD Pharmingen™, BD Biosciences, NJ, 562253 and 559763, respectively) according to the manufacturer's instructions. The stained cells were analyzed using the CytoFLEX S and CyExpert software (Beckman Coulter, CA).

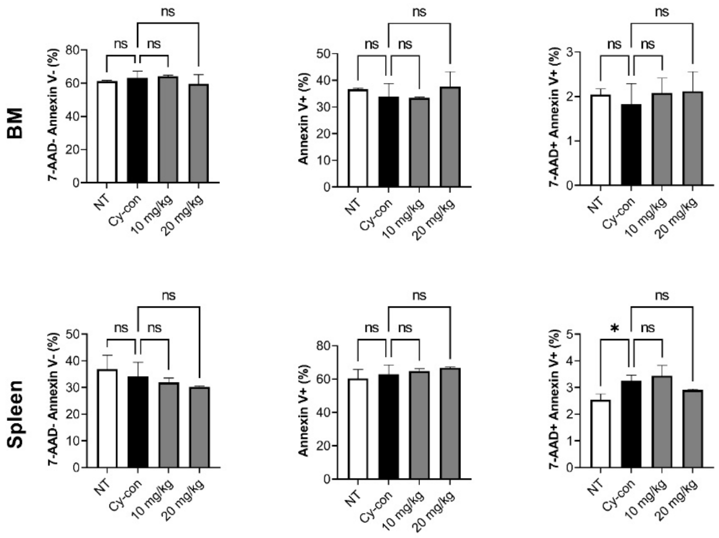

Figure S1. Effect of AGNEX on the induction of apoptosis in the BM cells and splenocytes (spleen) using cyclophosphamide. Splenocytes and BM cells were obtained from the bones of the hind limb and spleens of mice on the day of sacrifice. The percentages of Annexin V+ and 7-AAD+ cells were determined using 7-AAD and PE anti-Annexin V antibody staining. 7-AAD-/Annexin V- cells, Annexin V+ cells, and double positive cells represent healthy living cells, apoptotic cells, and dead cells, respectively. Values represent the mean ± SD. *$P < 0.05$, vs. Cy-con.

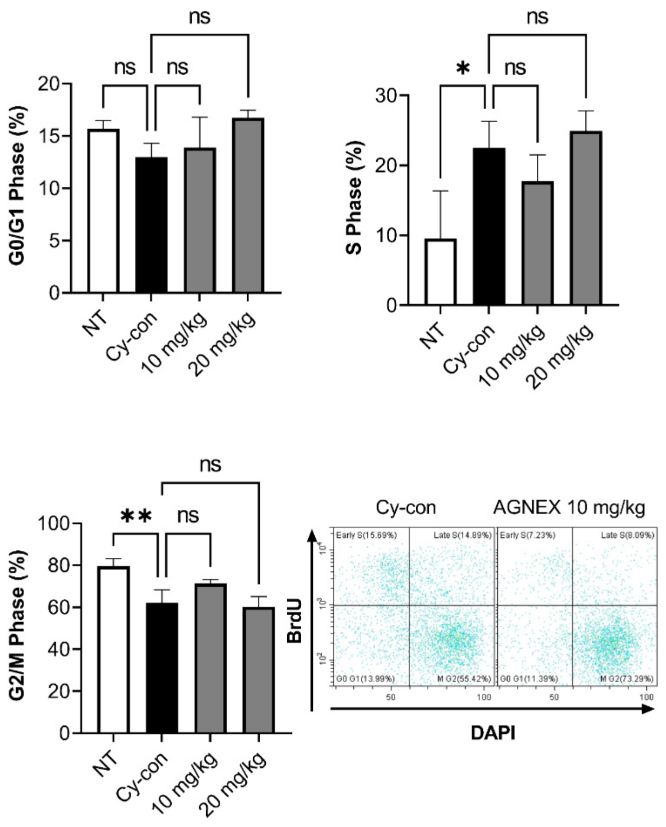

Figure S2. Confirmation of hematopoiesis by cell cycle analysis in splenocytes. Each group of mice received an intraperitoneal injection of BrdU (2 mg dissolved in 200 μl of PBS) 12 hours before sacrifice. Each cell cycle stage was stained using DAPI and PerCP-Cy5.5 anti-BrdU antibody. Percentages of each cell cycle fraction and representative results from Cy-con and AGNEX 10 mg/kg BM cells are shown. Each phase was determined by the signal intensities of BrdU and DAPI. The values represent the mean ± SD. **P* < *0.05*, ***P* < *0.01*, vs. Cy-con.

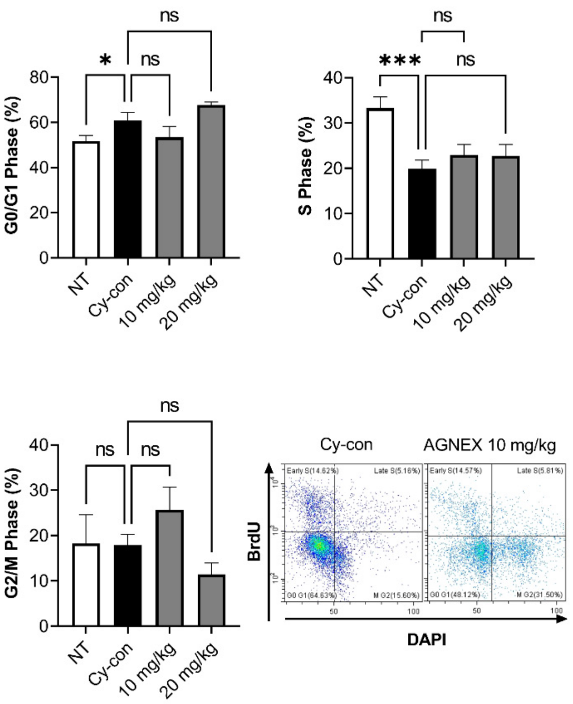

Figure S3. Confirmation of hematopoiesis by cell cycle analysis in the BM. Each group of mice received an intraperitoneal injection of BrdU (2 mg dissolved in 200 μl of PBS) 12 hours before sacrifice. Each cell cycle stage was stained using DAPI and PerCP-Cy5.5 anti-BrdU antibody. Percentages of each cell cycle fraction and representative results from Cy-con and AGNEX 10 mg/kg BM cells are shown. Each phase was determined by the signal intensities of BrdU and DAPI. Values represent the mean ± SD. *$P < 0.05$, ***$P < 0.001$, vs. Cy-con.

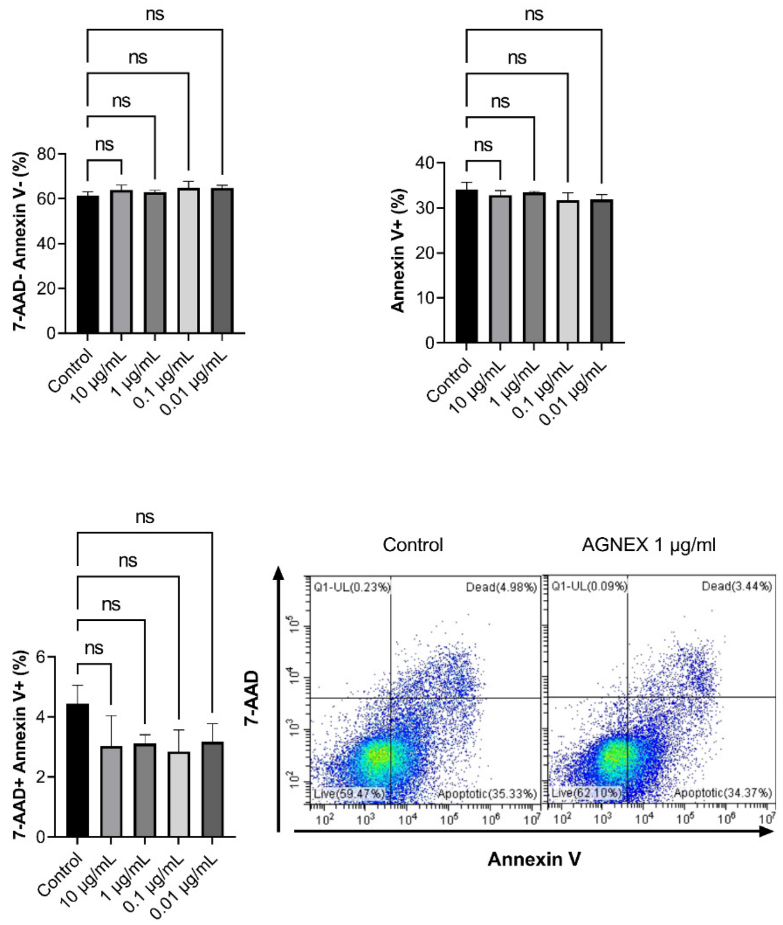

Figure S4. Effect of AGNEX on the induction of apoptosis in K562 cells using cyclophosphamide**.** K562 cells (1×10^6/well) were seeded and treated with cyclophosphamide at a concentration of 3.5 mg/mL and a varied concentration of AGNEX, a day before. The percentages of Annexin V+ and 7-AAD+ cells were determined using 7-AAD and PE anti-Annexin V antibody staining. 7-AAD-/Annexin V- cells, Annexin V+ cells, and double positive cells represent healthy living cells, apoptotic cells, and dead cells, respectively. Representative results from K562 cells treated with control (1% DMSO + 3.5 mg/mL cyclophosphamide) and AGNEX [1 μg/mL] (3.5 mg/mL cyclophosphamide + 1 μg/mL of AGNEX in 1% DMSO).

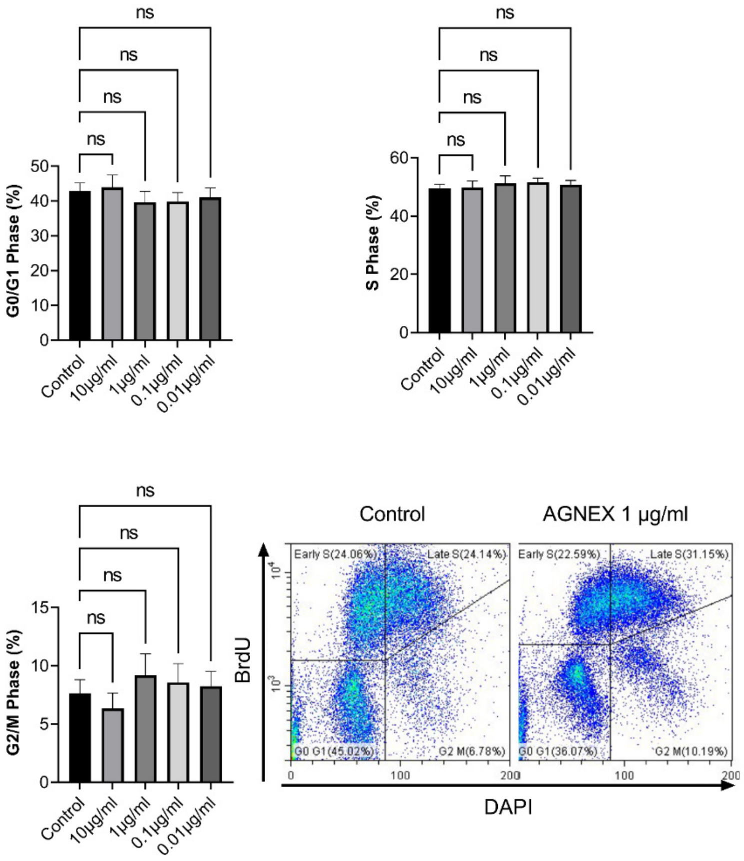

Figure S5. Cell cycle analysis of K562 cells cultured with varying concentrations of AGNEX**.** K562 cells (1×10^6/well) were seeded with AGNEX concentrations of 10, 1, 0.1, and 0.01 μg/mL. Each cell cycle stage was stained using DAPI and PerCP-Cy5.5 anti-BrdU antibody. Percentages of each cell cycle fraction and representative results from K562 cells treated with control (1%DMSO) and AGNEX [1 μg/mL] (1 μg/mL of AGNEX in 1% DMSO). Each phase was determined by the signal intensities of BrdU and DAPI.
